# Supplementary material for: Local unfolding of the HSP27 monomer regulates chaperone activity
Source: Nat Commun. 2019 Mar 6;10:1068. doi: 10.1038/s41467-019-08557-8 (PMC6403371; doi:10.1038/s41467-019-08557-8)
Supplement: Supplementary file 1 — Supplementary Information [file 41467_2019_8557_MOESM1_ESM.pdf]

Local unfolding of the HSP27 monomer regulates chaperone activity

T. R. Alderson *et al.*

p. 3 **Supplementary Fig. 1**: Analysis of NMR and mass spectra from full-length, oligomeric HSP27.
p. 4 **Supplementary Fig. 2**: Redox dependence of the aggregation of substrate proteins
p. 5 **Supplementary Fig. 3**: Characterisation of cHSP27 by NMR
p. 6 **Supplementary Fig. 4**: Characterisation of the H124K/C137S cHSP27 monomeric variant
p. 8 **Supplementary Fig. 5**: ps-ns dynamics in cHSP27 from ^{15}N relaxation
p. 10 **Supplementary Fig. 6**: μs-ms dynamics in cHSP27 from CPMG relaxation dispersion
p. 12 **Supplementary Fig. 7**: High-pressure NMR spectra of cHSP27
p. 14 **Supplementary Fig. 8**: Comparison of the cHSP27 monomer at pH 4.5 and pH 7
p. 16 **Supplementary Fig. 9**: Structural and dynamical comparison of the cHSP27 monomer and dimer
p. 18 **Supplementary Fig. 10**: αB-crystallin likely also partially unfolds in its monomeric form
p. 19 **Supplementary Table 1**: Thermodynamic and kinetic parameters from analysis of C137S NMR data
p. 20 **Supplementary Table 2**: ^{15}N $|\Delta\omega|$ values from CPMG RD analysis of C137S
p. 20 **Supplementary Table 3**: ^{15}N $|\Delta\omega|$ values from CPMG RD analysis of $L_{5,6+7}$ in oxidised cHSP27
p. 21 **Supplementary Table 4**: ^{15}N $|\Delta\omega|$ values from CPMG RD analysis of $\beta_{5,6+7}$ in oxidised cHSP27
p. 21 **Supplementary Table 5**: ^{15}N $|\Delta\omega|$ values from CPMG RD analysis of reduced cHSP27
p. 22 **Supplementary Table 6**: Primers for site-directed mutagenesis
p. 22 **Supplementary References**

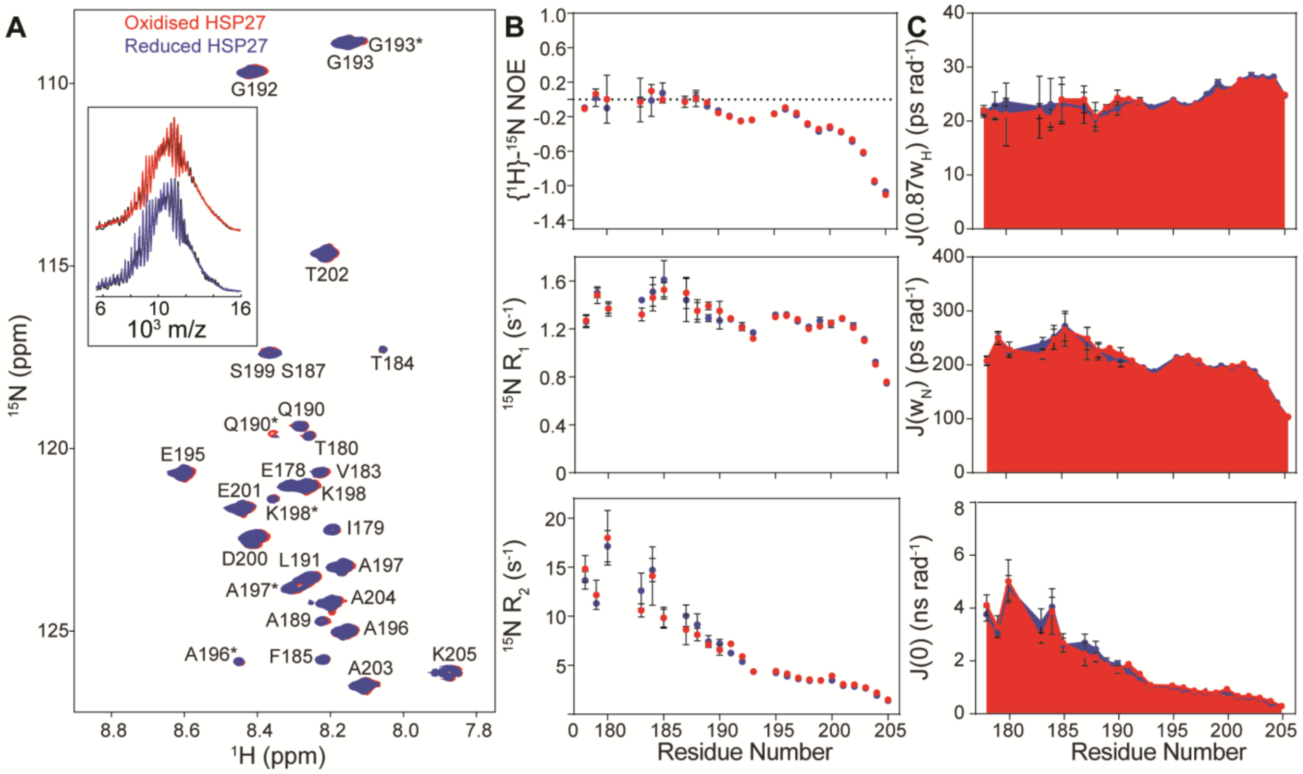

Supplementary Fig. 1: Analysis of NMR and mass spectra from full-length, oligomeric HSP27. (**A**) The C-terminal region (CTR) of HSP27 is sufficiently flexible to give rise to readily detectable resonances in solution-state NMR spectra[1,2]. Resonances in the 2D ^{1}H-^{15}N HSQC spectrum of oxidised (*red*) HSP27 at 2 mM monomer concentration in 30 mM NaH_2PO_4, 2 mM EDTA, 2 mM NaN_3, 100 mM NaCl, pH 7 at 25 °C can be assigned to residues E178-K205. Residues with asterisks arise from a minor state that has been ascribed to formation of a *cis*-G193-P194 peptide bond[1]. The addition of 5 mM BME (*blue*) had no discernible effect on either the chemical shifts of the resonances, nor their intensities. (*inset*) The native electrospray mass spectra of HSP27 in oxidised and reduced forms (Fig. 1) can be simulated (*black*) to estimate the underlying distribution of oligomers[3]. The spectra here can be well explained by assuming the oligomeric distribution follows a Gaussian distribution with means and standard deviations for (reduced/oxidised) of 23±3 / 27±3 and 7±2 / 8±2. Differences in the relative abundance of monomers and dimers cannot be readily distinguished by analysis of these data, but the spectra suggest that the higher-order oligomer distributions are very similar. (**B**) ^{15}N spin relaxation data collected on a 14.1 T NMR spectrometer at 25 °C. (*top*) {^{1}H}-^{15}N NOE, (*middle*) ^{15}N R_1, and (*bottom*) ^{15}N R_2 measurements[4,5] were acquired and reported here as a function of residue number and redox state. (**C**) Reduced spectral density functions were calculated from the ^{15}N relaxation data[6], yielding three frequencies of the spectral density function (*top*) $J(0)$, (*middle*) $J(\omega_N)$, and (*bottom*) $J(0.87\omega_H)$. No significant differences were observed in the spectral density functions between the two different redox states. It is likely that a combination of reduced motional freedom and chemical exchange result in the disappearance of residues prior to E178 [7,8,9].

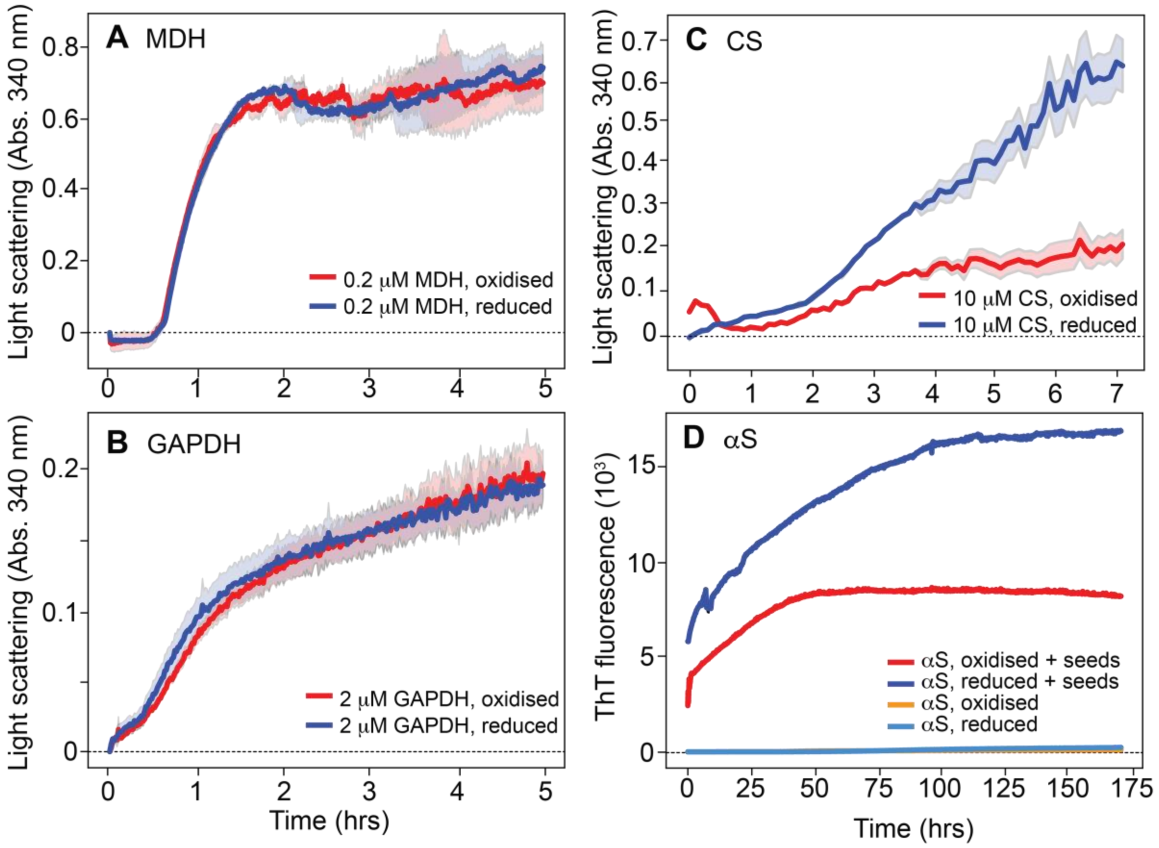

Supplementary Fig. 2: Redox dependence of the aggregation of substrate proteins. Amorphous aggregation monitored by light scattering at 340 nm versus time for (**A**) malate dehydrogenase (MDH), (**B**) glyceraldehyde-3-phosphate dehydrogenase (GAPDH), and (**C**) citrate synthase (CS). The concentrations of the substrates are indicated in the lower-right corner. *Red* traces are data recorded in the absence of reducing agent and *blue* traces correspond to data obtained in the presence of 5 mM reducing agent (DTT or BME). The buffers contained either 30 mM sodium phosphate, 3 mM EDTA at pH 7 (A, B) or 30 mM sodium phosphate, 3 mM EDTA, and 100 mM NaCl at pH 7 (C). Data were obtained at 40 °C (A, B) or 37 °C (C). (**D**) Fibril formation by α-synuclein (αS) over time monitored by ThT fluorescence at 485 nm following excitation at 440 nm. The concentration of αS was 200 μM and the buffer was phosphate buffered saline (PBS) at pH 7.4 with data obtained at 37 °C. In the traces indicated with "+ seeds", a small aliquot of pre-fibrillized seeds (200 μM initial concentration) was added. The samples contained either 5 mM DTT or no reducing agent. The addition of DTT increased the total fibril amount and diminished the initial rate of fibril formation, perhaps by altering solution conditions, e.g. ionic strength. For all data shown here, the solid lines represent the average of n = 3 (A, B, D) or n = 2 (C) with error bars representing the standard deviation from such measurements.

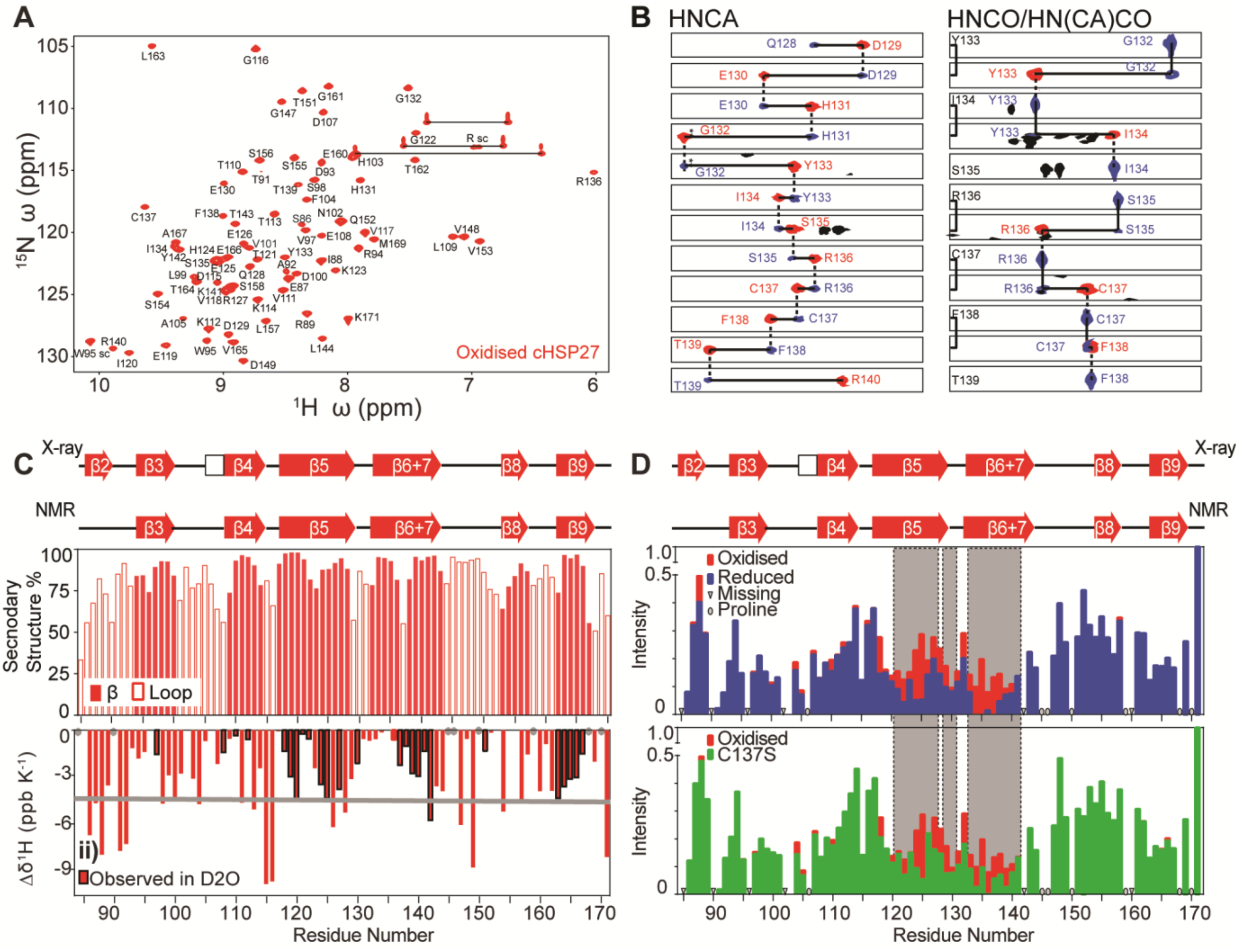

Supplementary Fig. 3: Characterisation of cHSP27 by NMR. (**A**) The assigned 2D ^{1}H-^{15}N HSQC spectrum of 1 mM [*U*-^{13}C,^{15}N]-oxidised cHSP27 at 25 °C, pH 7 in 30 mM NaH_2PO_4, 2 mM EDTA, 2 mM NaN_3. Horizontal lines indicate resonances originating from the same side-chain. (**B**) 2D ^{1}H-^{13}C strips taken from 3D HNCA (**i**) and HNCO/HN(CA)CO (**ii**) experiments recorded on [*U*-^{13}C,^{15}N]-oxidised cHSP27 at 1 mM used for resonance assignments[10]. The ^{15}N and ^{1}H chemical shifts are selected for specified residues (*i*, red *i-1* blue), allowing correlations in ^{13}C to define the sequential relationship between residues. The HNCA correlated *i* and *i*-1 residues, whereas the HNCO/HN(CA)CO pair report on *i*-1/*i* and *i*-1 connectivity. Strip plots for residues E130-T139 are shown, revealing unambiguous assignment. (**C**) (*top*) TALOS-N-derived secondary structure of oxidised cHSP27 using ^{1}H^{N}, ^{15}N, ^{13}Cα, ^{13}Cβ, and ^{13}CO chemical shifts. The secondary structure is displayed above in a ribbon format. The secondary structure of cHSP27 largely agrees with published crystal and solution structures[11,12]. We note that here the β2 strand near the N-terminus is disordered and the α-helix between the β3 and β4 strands resembles a loop, with some helical tendencies. (*bottom*) ^{1}H chemical shift temperature coefficients ($\Delta\delta^{1}H/\Delta T$) for oxidised cHSP27. Those below –4.6 ppb/K (*grey* line) indicate residues that are solvent-exposed and hydrogen bonded to water, whereas values above this are suggestive of hydrogen bonding to atoms within the protein[13]. Bars that are outlined in *black* are resonances that are highly protected from solvent, and remain observable after exchanging into 99.9% D_2O after 40 minutes (Observed in D_2O). Secondary structural elements and hydrogen bonding patterns are consistent with published structures[11,12]. (**D**) 2D ^{1}H-^{15}N HSQC spectra of 1 mM samples of oxidised (*red*), reduced (*blue*), or C137S (*green*) cHSP27 acquired in the presence of 5 mM β-mercaptoethanol (BME) (for the reduced cHSP27 sample only) and otherwise identical solution conditions (pH 7, 25 °C, buffer as in A). The relative HSQC peak intensities as a function of residue number for the three samples was determined, normalized to the C-terminus (K171), which was unaffected by changes in redox state. The significant attenuation of peak intensity in reduced cHSP27 and C137S could arise from either elevated solvent exchange or conformational exchange between two (or more) states on the μs-ms timescale. Elevated ^{15}N R_2 values in the in the region encompassing β5 and β6+7, and the loop between them, $L_{5,6+7}$ supports the latter (see Supplementary Figure 4).

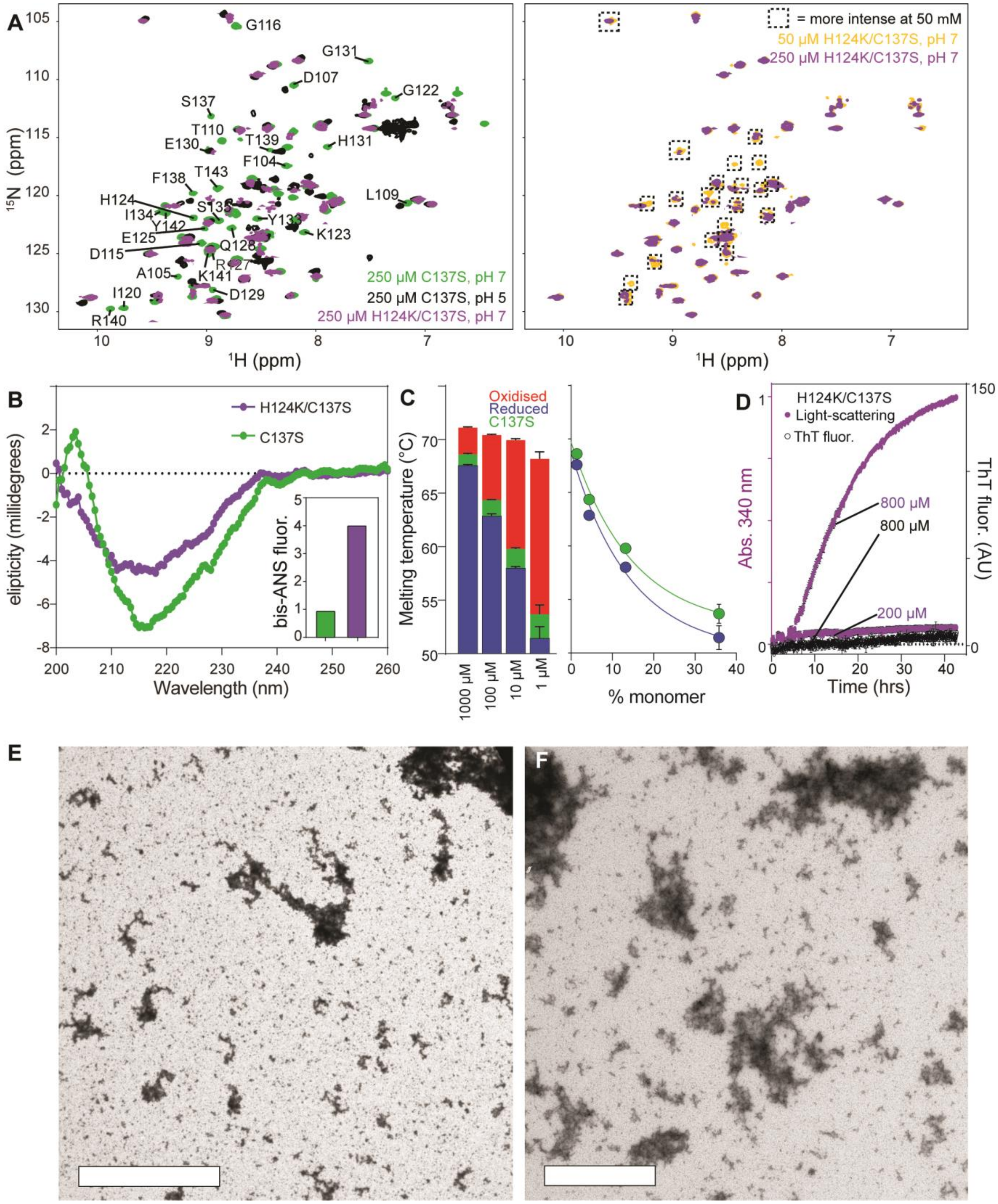

Supplementary Fig. 4: Characterisation of the H124K/C137S cHSP27 monomeric variant. The H124K/C137S monomeric mutant is destabilized and has a significantly heightened tendency to aggregate. (**A**) 2D ^{1}H-^{15}N HSQC spectra of C137S at 250 μM pH 7 where the protein is substantially dimeric (*green*) and pH 5 where it is predominately monomeric (*black*). The spectrum of H124K/C137S at pH 7 (*purple*) closely mimics the monomer, but many peaks are exchange-broadened and not visible. (*Right*) Decreasing the total concentration of H124/C137S from 250 μM to 50 μM at pH 7 significantly increases the relative signal intensities of many resonances, consistent with the stabilization of the monomeric state. (**B** *left*) CD spectra of cHSP27(H124K/C137S) and C137S at 20 μM and pH 7 reveal increased propensity for disordered conformations in the monomeric mutant. (*right*) Enhanced fluorescence in the presence of the dye bis-ANS indicates that the

H124K/C137S monomer has increased exposed hydrophobic sites with respect to C137S at pH 7. (**C**) The thermal stability of cHSP27 shown as a function of redox state and total protein concentration. (*left*) Nano-DSF was employed to measure the melting temperature (T_m) of oxidised (*red*), reduced (*blue*), and C137S (*green*) variants of cHSP27. The protein concentration varied logarithmically from 1000 μM to 1 μM. Data were measured in NMR buffer (methods) at pH 7 with 5 mM BME added to the reduced sample. Reduced and C137S cHSP27 showed a marked reduction in T_m at low concentration where monomers are favored. (*right*) The change in T_m is shown as a function of monomer population for reduced (*blue*) and C137S (*green*) cHSP27. A K_d of 0.4 μM is assumed in these calculations, which was the average values obtained from CPMG RD data sets measured at two different protein concentrations. The solid line depicts a fit to the equation exp(-k x). Error bars represent SD from the mean melting temperature derived from n = 3 replicates at each concentration. (**D**) Light scattering at 340 nm was monitored as a function of time to observe the self-aggregation of H124K/C137S at high concentration. At 800 μM, the double mutant is highly aggregation-prone, whereas the C137S is kinetically stable. By contrast, at 200 μM, the aggregation propensity of the double mutant is substantially reduced under these conditions (**A**, above). No significant fluorescence in the presence of ThioflavinT suggests that amorphous aggregates are present. Either n = 3 (800 μM) or n = 6 (200 μM) replicates were included, and the mean and 1 SD are reported. (**E, F**) Transmission electron microscopy (EM) images of amorphous aggregates of H124K/C137S. In both panels, the scale bar is shown in the lower left corner, representing 10 μm (*E*) or 1 μm (*F*)

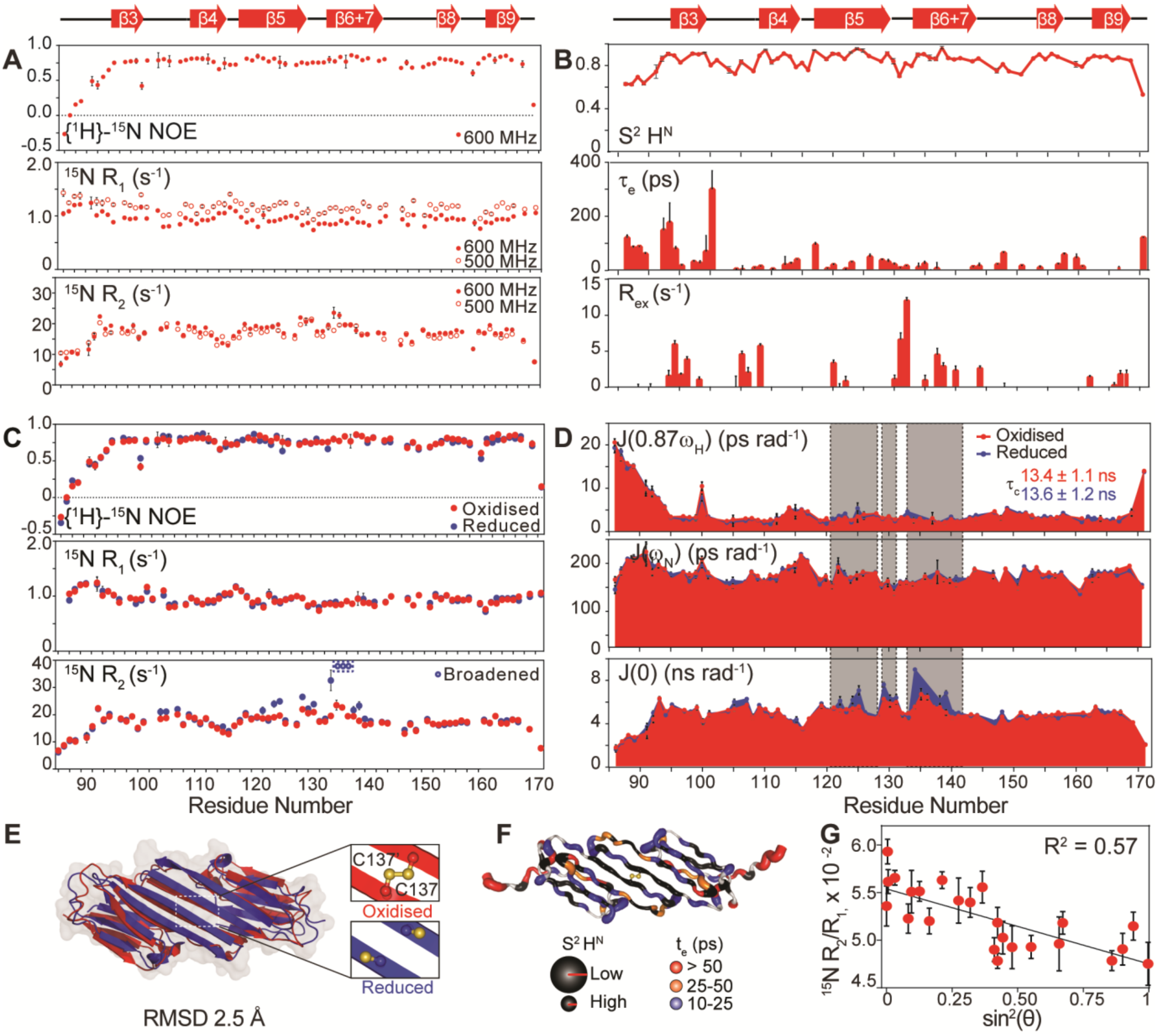

Supplementary Fig. 5: **ps-ns dynamics in cHSP27 from ^{15}N relaxation.** (**A**) Data acquired for a model-free analysis of ps-ns dynamics[14] in oxidised cHSP27. (*top*) {^{1}H}-^{15}N NOE recorded at 14.1 T (600 MHz ^{1}H Larmor frequency), (*middle*) ^{15}N R_1 data recorded at 11.7 T (500 MHz; open circles) and 14.1 T (closed circles), and (*bottom*) ^{15}N R_2 data recorded at 11.7 T and 14.1 T at 25 °C. (**B**) ^{15}N R_1, ^{15}N R_2, and heteronuclear NOE data acquired at 11.7 and 14.1 T were used to calculate (*top*) generalized order parameters (S^2), (*middle*) effective local correlation times (τ_e), and (*bottom*) exchange contributions to R_2 (R_{ex}) for backbone H–N bond vectors. For this analysis, the ^{15}N R_2/R_1 ratios from residues without evidence of fast internal motions or chemical exchange were first fitted to spectral density functions of varying rotational behaviour (isotropic, axially symmetric, and asymmetric) to determine the diffusion tensor of cHSP27 and the global rotational correlation time (τ_c)[15]. The usage of an axially symmetric diffusion tensor (D_{par}/D_{perp} = 1.52) was identified by comparing fits using a statistical F-test[15]; the determined τ_c was 13.65 ns (see **G**). The software ModelFree4.1[16] was used with an H–N bond vector length of 1.02 Å and a ^{15}N CSA of –160 ppm. The analysis reveals the protein to be generally very rigid, with some relatively rapid motion towards the disordered N-terminus. Exchange contributions were found to be significant in the region of $L_{5,6+7}$. (**C**) Comparison of ^{15}N relaxation measurements on 1 mM [U-^{13}C,^{15}N]-oxidised (*red*) and reduced (*blue*) cHSP27. (*top*) {^{1}H}-^{15}N NOE, (*middle*) ^{15}N R_1, and (*bottom*) ^{15}N R_2. All data were recorded at 14.1 T and 25 °C. The open circles in the bottom panel correspond to resonances that were too broad to accurately quantify R_2 rates, suggestive of chemical exchange on the μs-ms timescale in this region (β6+7 strand). (**D**) Calculation of reduced spectral density functions[6] from the data in panel (C) enables a comparison of fast (ps–ns) and slow (μs–ms) dynamics in reduced and oxidised cHSP27, and provides the spectral density function at three frequencies, (*top*) $J(0.87\omega_H)$, (*middle*) $J(\omega_N)$, and (*bottom*), $J(0)$. The ps-ns dynamics were highly similar in the two forms of the protein. The β5 and β6+7 strands and the loop between them ($L_{5,6+7}$), indicated by

a *grey* box, show large peak intensity attenuations upon reduction, indicative of either elevated solvent exchange or chemical exchange with μs-ms timescale dynamics. The elevated ^{15}N R_2 rates for residues with attenuated intensities supports the latter option. (**E**) Crystal and NMR structures of reduced (*blue*, PDB 4mjh) and oxidised (*red*, PDB 2n3j) cHSP27 dimers are highly similar (backbone RMSD of 2.5 Å). (**F**) S^2 and τ_e values are mapped onto the structure of oxidised cHSP27 (PDB 2n3j). Low (high) S^2 values are encoded by a large (small) tube radius and τ_e values are indicated by a color gradient from *red* (high) to *blue* (low). (**G**) ^{15}N R_2/R_1 ratios for oxidised cHSP27 plotted as a function of the angle between a given N-H bond vector and the principal component of the cHSP27 diffusion tensor. These calculations used the X-ray structure of cHSP27 (PDB 4mjh), and the parameters were used for the local correlation time calculation (see B). All error bars are SD values derived from fitting procedures.

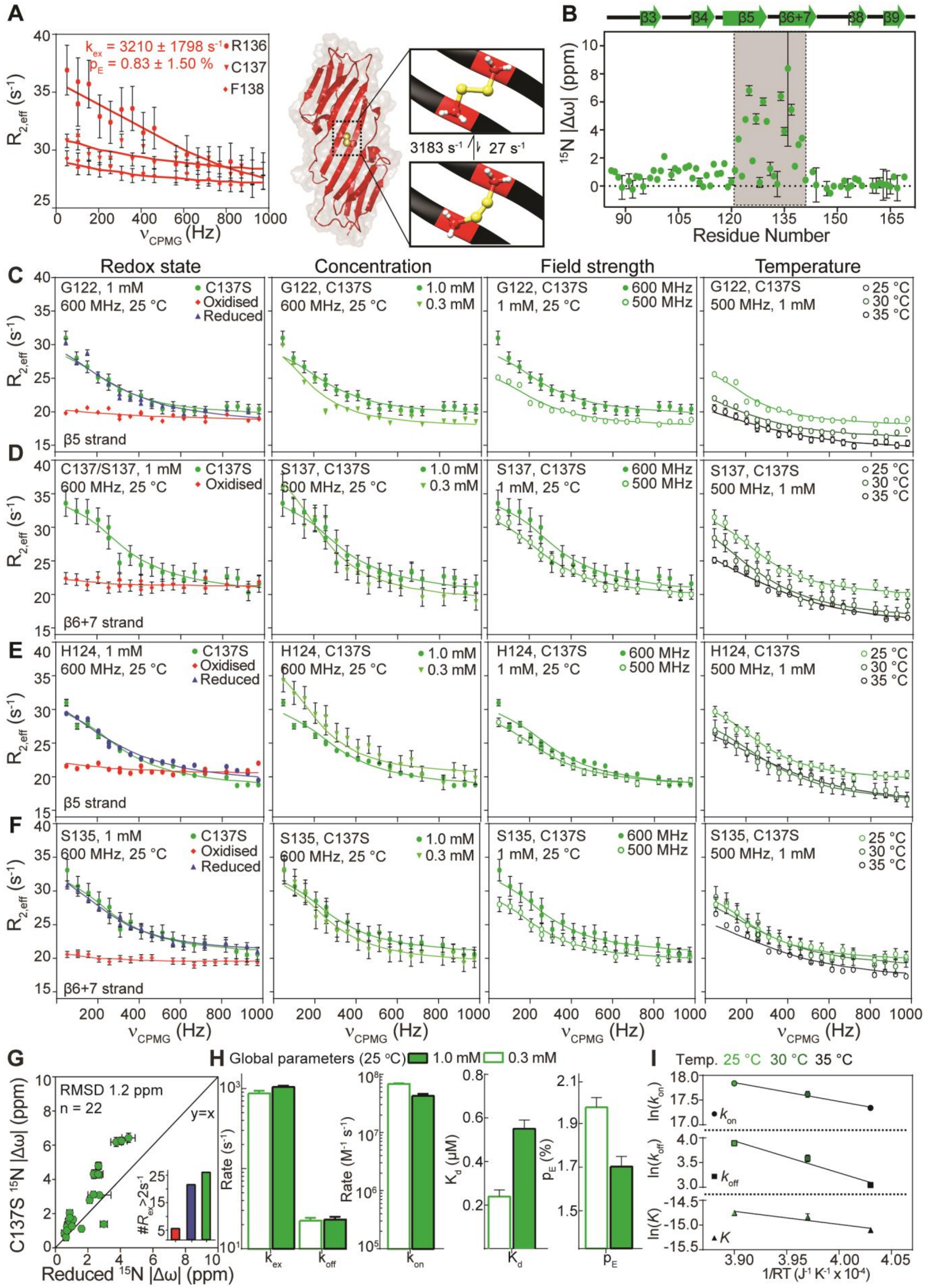

Supplementary Fig. 6: µs-ms dynamics in cHSP27 from CPMG relaxation dispersion. ^{15}N CPMG RD data were acquired at 11.7 and 14.1 T over a range of temperatures (25, 30, 35 °C) and concentrations (0.3 and 1 mM).

CPMG RD curves were globally fit to a model of two-site chemical exchange using CATIA[17]. (**A**) In a 1.5 mM sample of oxidised cHSP27, residues R136, C137, and F138 yielded ^{15}N CPMG RD curves that were not well explained by the thermodynamic parameters used to fit the group of residues in $L_{5,6+7}$. Analysing R136, C137, and F138 as a group yielded k_{ex} = ~3200 s^{-1} with p_E = ~1 %. Such motions likely arise from *gauche-trans* rotations of the inter-molecular disulphide bond[18]. (**B**) The ^{15}N chemical shift differences from C137S obtained from a global analysis of ^{15}N CPMG RD data at 25 ^{o}C. (**C-F**) ^{15}N CPMG RD data for G122 (**C**), C137/S137 (**D**), H124 (**E**) and S135 (**F**) in oxidised (*red*), reduced (*blue*) and C137S (*green*) cHSP27. Note that the resonance for C137 in the reduced state is too broad to yield reliable measurements. These residues are representative of the major secondary structural elements (β5 and β6+7 strands). Residues in the loop between them ($L_{5,6+7}$) are shown in Fig. 4. CPMG RD curves at 600 MHz, 1 mM and 25 ^{o}C The first column (Redox State) reveals the similarities in dynamics between reduced cHSP27 and C137S. The second column (Concentration) reveals the variation of the dispersions from C137S with concentration (1 mM and 0.3 mM). The third column (Field Strength) shows the static magnetic field dependence of dispersions from C137S (11.7 and 14.1 T). The final column (Temperature) shows variation in the dispersions from C137S with temperature (25, 30, and 35 ^{o}C). These data were analysed globally using CATIA[17,19] to simultaneously provide local chemical shift differences and global thermodynamic and kinetic parameters. (**G**) Correlation between CPMG RD-derived ^{15}N chemical shift changes for C137S and reduced cHSP27. The RMSD and number of residues are listed. Note that CPMG RD data on reduced cHSP27 were available at only one magnetic field strength (14.1 T), whereas two magnetic field strengths were used to fit the C137S data (14.1, 11.7 T). However, the overall similarity between the two datasets (G, C-F) confirms that C137S mimics the reduced state. *Inset*: the number of residues with R_{ex} > 2 s^{-1} at 14.1 T, where R_{ex} is the difference in $R_{2,eff}$ at the lowest and highest CPMG pulse frequencies. (**H**) Kinetic parameters derived from the concentration-dependent CPMG RD data shown above for C137S. k_{on} refers to the association rate of monomers (M^{-1} s^{-1}) and k_{off} to the dissociation rate of C137S dimers (s^{-1}) at 25 ^{o}C. See the text below for mathematical descriptions of their derivations. (**I**) Temperature variation in the kinetic and thermodynamic parameters reveals activation and thermal properties of the exchange process.

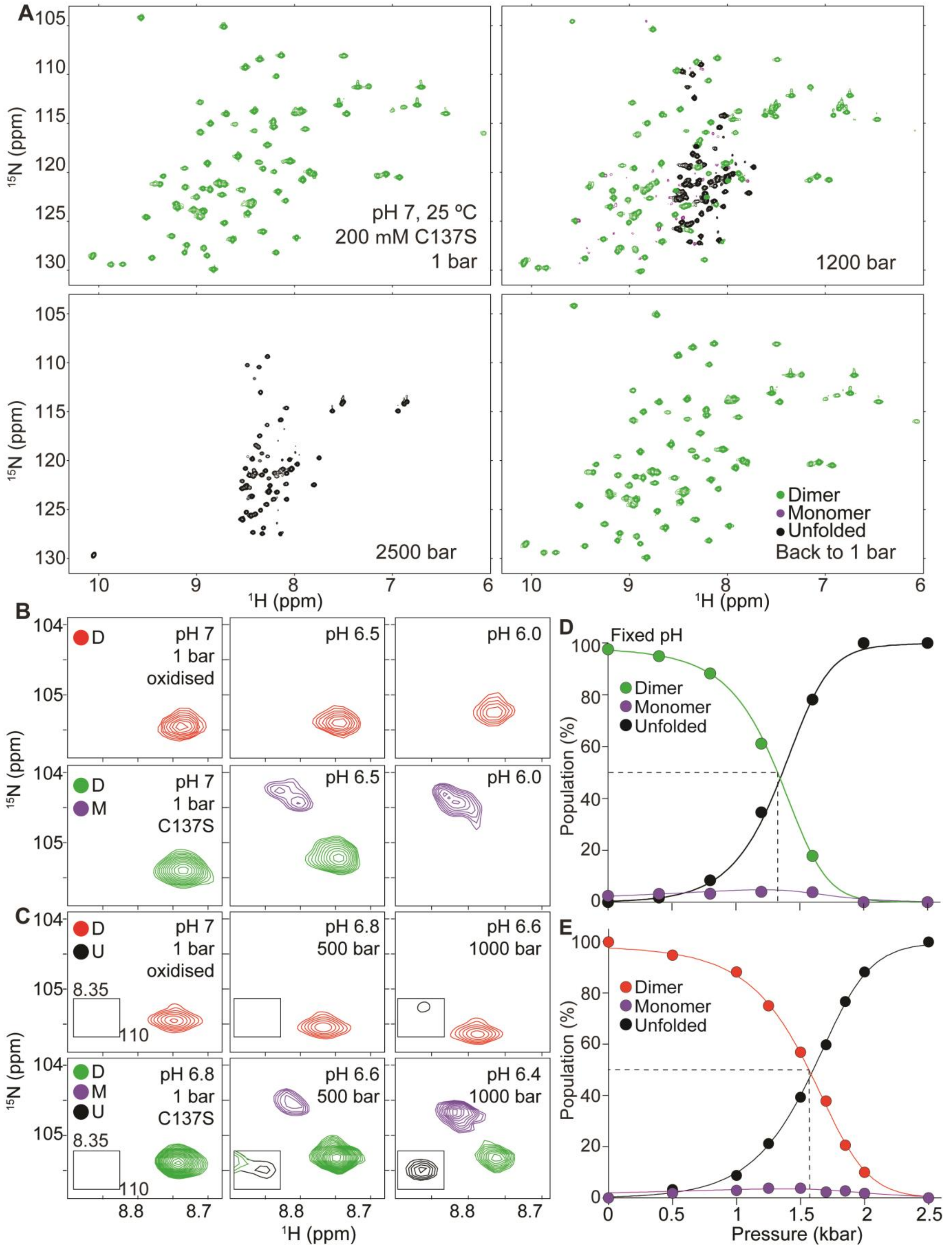

Supplementary Fig. 7: High-pressure NMR spectra of cHSP27. (**A**) 2D ^{1}H-^{15}N HSQC spectra of [*U*-^{2}H,^{13}C,^{15}N]-C137S at 200 μM, pH 7, 25 °C, as a function of hydrostatic pressure. Peaks from the dimer are colored *green*, the monomer *purple*, and the unfolded monomer *black*. Minor peaks from the intermediate monomeric state are evident in the 1200 bar spectrum. The pressure-induced unfolding of C137S is reversible, as

demonstrated by the recovery of the folded spectrum at 1 bar after 2500 bar. To ensure that no pH changes occurred with pressure, a baroresistant buffer was prepared[20], using a mixture of 100 mM Tris-HCl and 100 mM phosphate buffer at pH 7. This Tris/phosphate buffer is insensitive to changes in pH with pressure. The standard NMR buffer used throughout this work (30 mM sodium phosphate, 2 mM EDTA, pH 7) experiences a change in pH of –0.9 units between 1 bar and 2500 bar due to the change in the pK_a of phosphate with pressure. The minor peaks in the spectrum at 2500 bar arise from *cis*-Pro formation at the seven X-Pro bonds in C137S.[21] (**B**) Zoomed-in region from panel (A) showing the multiple resonances from G116: monomer (G116M), dimer (G116D), and unfolded (G116U). The first row depicts data from oxidised cHSP27 and the second row from C137S as a function of pH. (**C**) The same as (B) but as a function of pressure in phosphate buffer where the pH varies with pressure. (**D**) Three-state fit to the data recorded as a function of pressure at a constant pH value. See Figure 5b for the same fit performed on a sample in phosphate buffer, which varies pH with pressure. (**E**) Same as (D), but for oxidised cHSP27 in phosphate buffer.

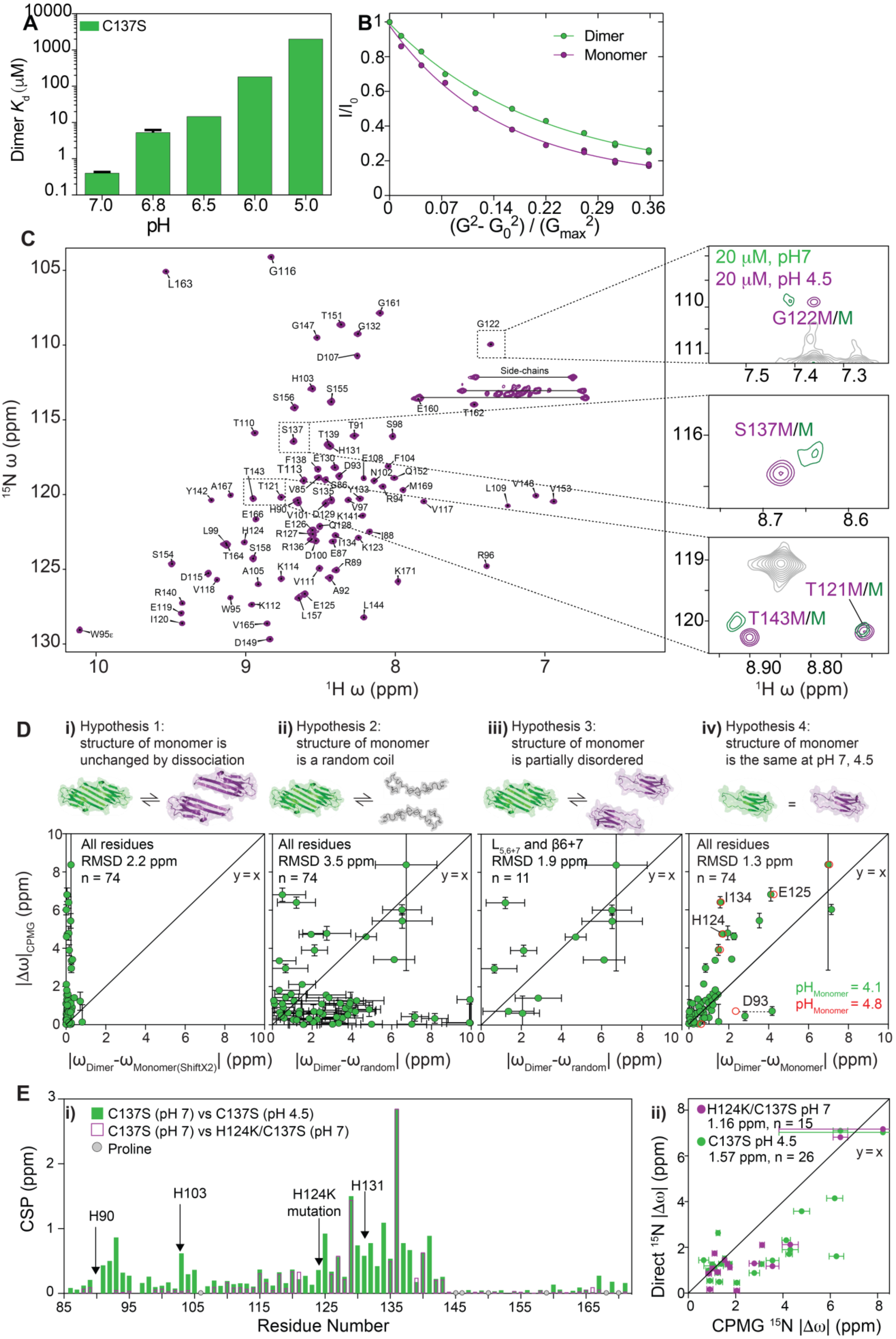
A
C137S
Dimer K_d (μM)
pH
7.0
6.8
6.5
6.0
5.0
B
Dimer
Monomer
I/I_0
$(G^2- G_0^2) / (G_{max}^2)$
C
^{15}N ω (ppm)
^{1}H ω (ppm)
Side-chains
20 μM, pH7
20 μM, pH 4.5
G122M/M
S137M/M
T121M/M
T143M/M
D
i) Hypothesis 1: structure of monomer is unchanged by dissociation
ii) Hypothesis 2: structure of monomer is a random coil
iii) Hypothesis 3: structure of monomer is partially disordered
iv) Hypothesis 4: structure of monomer is the same at pH 7, 4.5
All residues
RMSD 2.2 ppm
n = 74
All residues
RMSD 3.5 ppm
n = 74
$L_{5,6+7}$ and β6+7
RMSD 1.9 ppm
n = 11
All residues
RMSD 1.3 ppm
n = 74
y = x
$|\Delta\omega|_{CPMG}$ (ppm)
$|\omega_{Dimer}-\omega_{Monomer(ShiftX2)}|$ (ppm)
$|\omega_{Dimer}-\omega_{random}|$ (ppm)
$|\omega_{Dimer}-\omega_{Monomer}|$ (ppm)
$pH_{Monomer}$ = 4.1
$pH_{Monomer}$ = 4.8
I134
E125
H124
D93
E
i)
C137S (pH 7) vs C137S (pH 4.5)
C137S (pH 7) vs H124K/C137S (pH 7)
Proline
CSP (ppm)
Residue Number
H90
H103
H124K mutation
H131
ii)
H124K/C137S pH 7
1.16 ppm, n = 15
C137S pH 4.5
1.57 ppm, n = 26
y = x
Direct ^{15}N |Δω| (ppm)
CPMG ^{15}N |Δω| (ppm)

Supplementary Fig. 8: Comparison of the cHSP27 monomer at pH 4.5 and pH 7. **(A)** The K_d for C137S dimer dissociation shown as a function of pH. Note that the K_d is raised by over four orders of magnitude upon lowering the pH from 7 to 5. (**B**) NMR pulsed field gradient (PFG)-based translational diffusion measurements on the C137S dimer at pH 7 (*green*) and monomer at pH 4.1 (*purple*) confirm that the sample at acidic pH is indeed monomeric. The diffusion constant for the monomeric sample is *ca.* 25% larger than the dimer, consistent with theoretical expectations. The y-axis contains the decay of signal intensity (I/I_0) and the x-axis depicts the gradient strength. (**C**) Assigned 2D ^{1}H-^{15}N HSQC spectrum of the C137S monomer at pH 4.5 and 20 μM total protein concentration. The sample aggregates under acidic conditions at elevated protein concentration. Boxed regions contain zoomed-in views of the spectrum of the C137S monomer (*purple*) and dimer (*green*) at identical total protein concentration. Minor peaks from the C137S monomer at pH 7 are observable under these conditions, as the monomer-dimer equilibrium exists in slow exchange. The close correspondence between C137S monomer resonances at pH 7 and pH 4.5 indicates that the overall structures are similar. The grey resonances arise from the major dimer peaks in the pH 7 sample. (**D**) To identify the conformation of the dissociated, free monomer, four hypotheses were tested by comparing the expected (ShiftX2 [22]) or measured (experimental) chemical shifts to those obtained from CPMG measurements under physiological conditions (Fig. 4, Supplementary Fig. 6). Although not all residues were used in the global fits to derive thermodynamic and kinetic parameters (see Methods), the k_{ex} and p_E values derived from this analysis were fixed to generate ^{15}N chemical shift changes for all residues in the monomer. Thus, ^{15}N $|\Delta\omega|$ values were calculated for all residues (n = 74) using data acquired at multiple fields at 25 °C and 1 mM protein concentration (Supplementary Fig. 6). The $|\Delta\omega|$ values for residues not included in the global analysis were found to small and poorly defined, hence their clustering near 0 ppm. Hypothesis 1: the conformation of the monomer is essentially identical to its conformation in the dimer. This corresponds to a rigid body dimerization event. To test this hypothesis, experimental ^{15}N chemical shift changes between the dimer and monomer were calculated using ShiftX2 [22] for the monomer (chain A from PDB 4mjh) and dimer (PDB 4mjh). The correlation between CPMG RD-derived chemical shifts and this prediction was poor, indicating that the monomer does not entirely resemble its conformation in the dimer. Hypothesis 2: the conformation of the monomer is fully unfolded. The ^{15}N chemical shift changes expected for a transition from the C137S dimer to a random coil were predicted via the ncIDP[23] webserver and compared to the experimentally determined CPMG $|\Delta\omega|$ values. The correlation between is relatively poor, indicating that the monomer retains (at least some) structured elements. Hypothesis 3: the conformation of the monomer is partially unfolded in the regions $L_{5,6+7}$ and β6+7. Similar to hypothesis 2, ncIDP ^{15}N chemical shift changes were correlated with experimentally determined CPMG values for the region indicated. This reasonable correlation indicates that this region of C137S has likely unfolded in its monomeric state. Hypothesis 4: the conformation of the monomer at pH 7 is similar to the species directly observed at acidic pH and dilute protein concentrations. The reasonable correlation between the CPMG chemical shift changes and those measured directly from the stabilized intermediate supports this hypothesis. In addition, the observed chemical shifts of the monomer at pH 7 (see A, above) further support this notion. Some of the outliers on this correlation plot are due to ionization at low pH, as revealed by some ^{15}N chemical shift changes between pH 4.1 (*green*) and pH 4.8 (*red*) (e.g. D93). Only the handful of resonances that showed ^{15}N chemical shift changes between pH 4.1 and pH 4.8 are indicated here. As compared to pH 7, His residues (pK_a ~ 6) will become protonated and Asp/Glu residues will depend on their local pK_a values (pK_a ~3.8-4.1). The resonance from D93 shows a large, pH-dependent ^{15}N chemical shift change in the 4.1 – 4.8 range. Other residues near His (e.g. H90, H103, H124, H131) may show ^{15}N chemical shift changes closer to pH 6 and above. (**E**) Comparison of the C137S dimer at pH 7 to the C137S monomer at pH 4.5 and the H124K/C137S monomer at pH 7. (*left*) Combined, weighted ^{1}H and ^{15}N CSPs between (*green*) the C137S dimer at pH 7 and C137S monomer at pH 4.5 and (*purple*) the C137S dimer at pH 7 and H124K/C137S monomer at pH 7. Resonance assignments for the H124K/C137S monomer were obtained by transferring those from the fully assigned C137S monomer at pH 4.5, yielding 56 assignable resonances. In general, the pattern of CSPs between the C137S dimer and H124K/C137S monomer resemble those for the C137S dimer (pH 7) and C137S monomer (pH 4.5). Differences are observed near H90, H103, and H124, which either have fully protonated side-chains that affect the backbone N-H chemical shift (H90, H103) or have been mutated and thus resonate in a new position (H124). (*right*) Comparison of ^{15}N $|\Delta\omega|$ values measured on C137S by CPMG RD, which report on monomer-dimer structural transitions, and ^{15}N $|\Delta\omega|$ values between the C137S dimer and the C137S monomer at pH 4.5 (*green*) or H124K/C137S monomer at pH 7 (*purple*). The directly observed values were obtained through comparison of HSQC spectra. The RMSD and number of analysed residues are indicated in the legend.

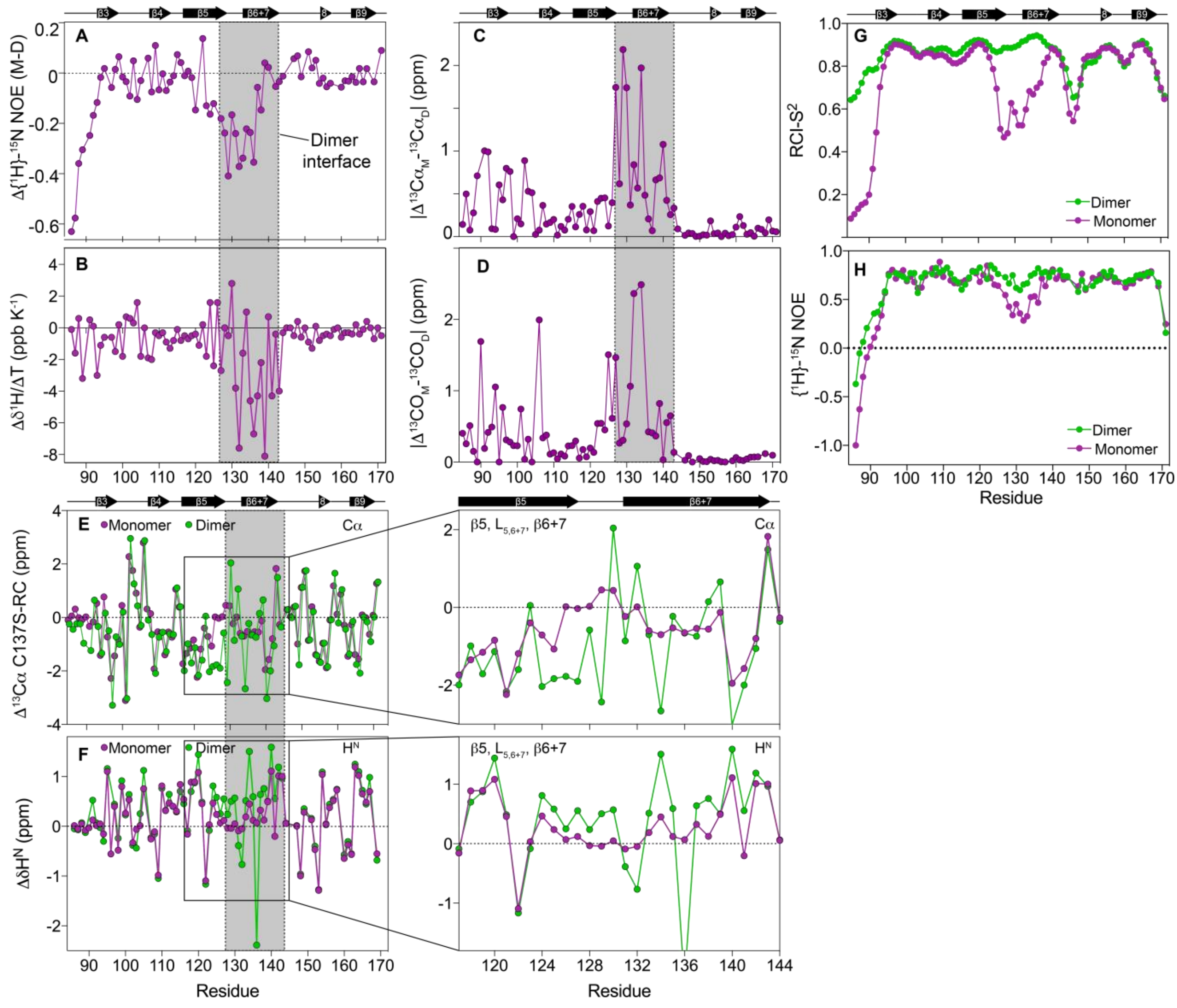

Supplementary Fig. 9: Structural and dynamical comparison of the cHSP27 monomer and dimer. (**A**) The difference in {^{1}H}-^{15}N NOE values between the C137S monomer and dimer (monomer-dimer). Negative values indicate enhanced backbone dynamics in the monomeric state. (**B**) The difference in ^{1}H chemical shift temperature coefficients ($\Delta\delta^1$H/ΔT) for the C137S monomer and dimer (monomer-dimer). More negative values indicate a progressively lower likelihood that a given amide proton is involved in a hydrogen bond to other atoms in the protein[13]. The region that comprises the dimer interface shows many residues with a lower likelihood of hydrogen bond formation, consistent with the less secondary structure. Residues G147-K171 show very little differences between the monomer and dimer. (**C**) and (**D**) show the absolute value of the difference in ^{13}Cα (C) and ^{13}CO (D) chemical shifts for the C137S monomer and dimer. These nuclei are particularly sensitive to backbone conformations, and large differences reflect structural changes. The region comprising the dimer interface and residues near H90 and H103 show the largest changes. From G147 to K171, there are essentially no structural differences between the C137S monomer and dimer, indicating that the dimeric conformation is preserved in this region. Notably, these residues yield small ^{15}N $|\Delta\omega|$ values in the CPMG RD experiments at pH 7, and therefore they comprise the residues that cluster between ~0.5 and 0 ppm in Supplementary Fig. 6. (**E**, **F**) Secondary chemical shifts ($\Delta\delta$), or the difference between the measured chemical shift for a given nucleus within a residue and the same nucleus in a random coil conformation, are shown for ^{13}Cα and ^{1}H^N. A value of zero indicates a random coil conformation, and positive or negative values indicate secondary structure, with the sign and magnitude depending on the nucleus and secondary structure element. The zoomed-in regions show the β5 strand, $L_{5,6+7}$, and β6+7 strand, note the small $\Delta\delta$ values near the end of β5, throughout $L_{5,6+7}$, and up to F138/T139 in β6+7. Secondary structure motifs of the cHSP27 dimer, as determined from PDB 4mjh, are shown above for reference. (**G**) RCI-determined N-H S^2 values for the C137S dimer (*green*) and monomer (*purple*). (**H**) {^{1}H}-^{15}N

NOE values for the C137S monomer (*purple*) and dimer (*green*). The data in Figure 7 b are truncated at 0, whereas here the full data are shown.

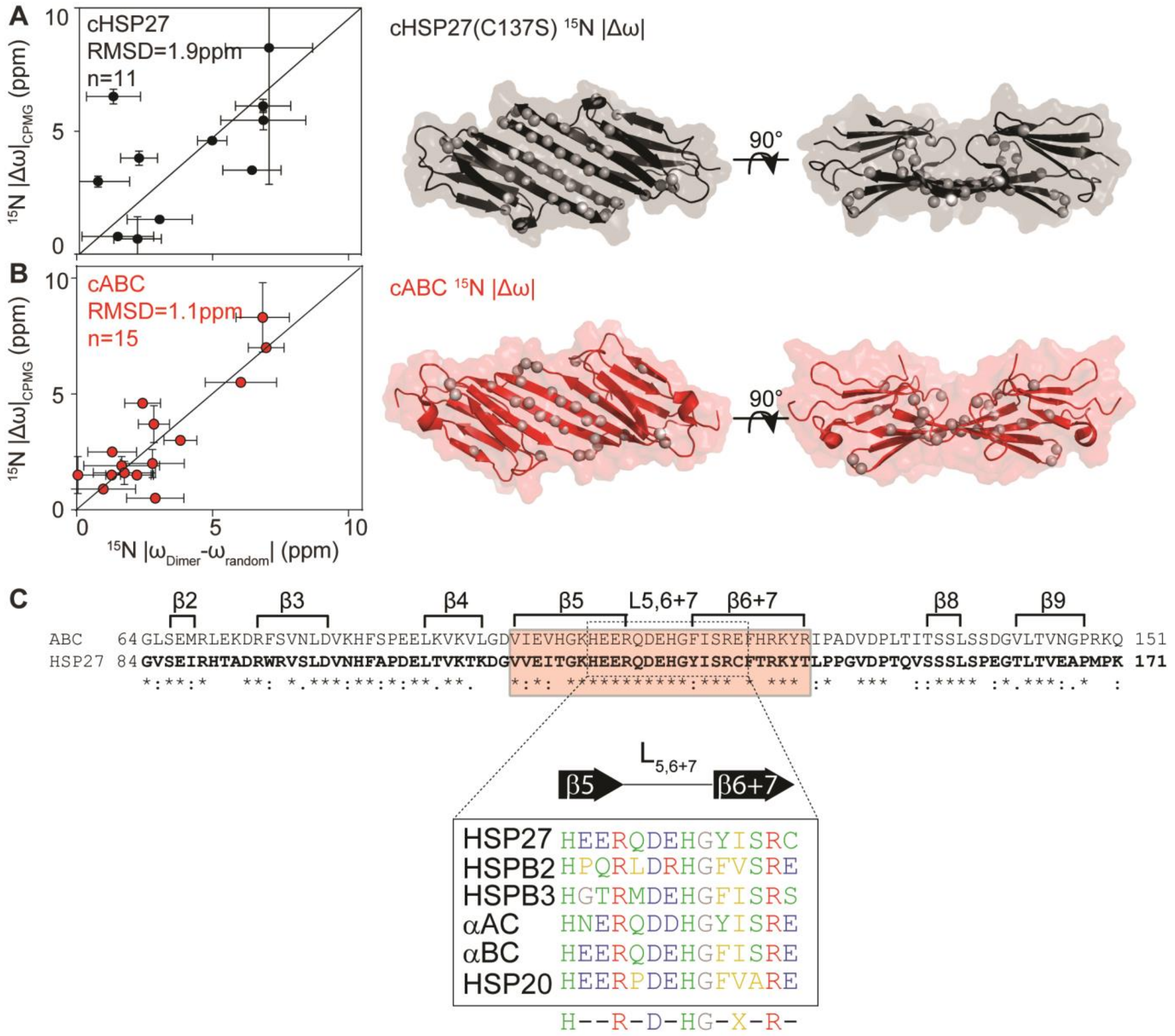

Supplementary Fig. 10. αB-crystallin (ABC) likely also partially unfolds in its monomeric form. (**A/B**) ^{15}N chemical shift changes that were measured by CPMG (C137S and cABC) or directly from a pH titration (cABC) are correlated with expected ^{15}N chemical shift changes upon the formation of random coil conformations. The ncIDP database was used to calculate sequence-corrected ^{15}N random coil chemical shifts[23], shown here as the mean ± 1 SD. The agreement between experimentally measured ^{15}N chemical shift changes upon monomerization with chemical shift changes expected for random coil conformations is reasonable (C137S RMSD: 1.6 ppm, n = 14; cABC RMSD: 1.1 ppm, n = 15), suggesting the change in conformation reflects the formation of a disordered monomer for both cHSP27 (A) and αB-crystallin (B). The locations of the residues that show significant relaxation dispersion curves are indicated (cHSP27 PDB 4mjh, *black*; cABC PDB 4m5s, *red*). Data from αB-crystallin were extracted from Rajagopal *et al.*[24] and originate from ^{15}N CPMG RD data (residues 102, 106, 109, 117, 118, and 136) and ^{15}N chemical shift changes measured in a pH titration (residues 104, 108, 113, 114, 115, 116, 120, 121, and 126). (**C**) Amino acid sequence comparison between the ACDs of αB-crystallin and HSP27. The β-strands are indicated, and residues in the β5 and β6+7 strands are indicated (box). There is significant sequence conservation in this area. The inset depicts the sequence alignment of human sHSPs HSPB1-HSPB6 (i.e. HSP27, HSPB2, HSPB3, αA-crystallin, αB-crystallin, and HSP20) in the region β5-$L_{5,6+7}$-β6+7.

Experiment	Parameter	pH	Temp. (°C)	Concentration *1.0 mM*	*0.3 mM*
**A)** ^{15}N CPMG	k_{ex} (s^{-1})	7	25<br>30<br>35	1053 ± 48<br>1991 ± 136<br>2451 ± 117	888 ± 51
**A)** ^{15}N CPMG	p_E (%)	7	25<br>30<br>35	1.70 ± 0.04<br>1.84 ± 0.06<br>1.83 ± 0.06	1.95 ± 0.04
**A)** ^{15}N CPMG	k_{eg} (s^{-1})	7	25<br>30<br>35	1035 ± 47<br>1954 ± 133<br>2406 ± 115	870 ± 50
**A)** ^{15}N CPMG	k_{on} ($M^{-1}s^{-1}$)	7	25<br>30<br>35	3.0 ± 0.2 x 10^7<br>5.3 ± 0.4 x 10^7<br>6.6 ± 0.5 x 10^7	7.4 ± 0.2 x 10^7
**A)** ^{15}N CPMG	k_{off} (s^{-1})	7	25<br>30<br>35	17.9 ± 0.8<br>36.6 ± 2.5<br>44.9 ± 2.5	17.3 ± 1.4
**A)** ^{15}N CPMG	K_d^{CPMG} (μM)	7	25<br>30<br>35	0.59 ± 0.02<br>0.69 ± 0.04<br>0.68 ± 0.04	0.23 ± 0.03
**B)** ^{15}N CPMG	ΔH (kJ mol^{-1})<br>ΔS (J mol^{-1} K^{-1})	7	25/30/35	26 ± 8<br>60 ± 27	
**B)** ^{15}N CPMG	ΔH_{eg} (kJ mol^{-1})<br>ΔS_{eg} (J mol^{-1} K^{-1})	7	25/30/35	40 ± 4<br>80 ± 14	
**B)** ^{15}N CPMG	ΔH_{ge} (kJ mol^{-1})<br>ΔS_{ge} (J mol^{-1} K^{-1})	7	25/30/35	66 ± 4<br>140 ± 14	
**C)** HSQC versus conc.	K_d^{HSQC} (μM)	7	25	0.7 ± 0.1	
**C**) HSQC versus pH	K_d^{HSQC} (μM)	7<br>6.8<br>6.5<br>6<br>5	25<br>25<br>25<br>25<br>25	0.3 ± 0.1<br>6.5 ± 0.9<br>14.6 ± 2<br>183 ± 20<br>> 2000	
**D)** HSQC versus pressure	ΔV_{A2toA} (mL mol^{-1})	6.8*<br>7<br>7, ox	25<br>25<br>25		-63 ± 4<br>-32 ± 15<br>-42 ± 13
**D)** HSQC versus pressure	ΔV_{AtoB} (mL mol^{-1})	6.8*<br>7<br>7, ox	25<br>25<br>25		-54 ± 3<br>-77 ± 12<br>-62 ± 6
**D)** HSQC versus pressure	K_U^0	6.8*<br>7	25<br>25		0.04 ± 0.02<br>0.13 ± 0.01
**D)** HSQC versus pressure	K_D^0 (μM)	6.8*<br>7	25<br>25		6.5 ± 0.9<br>0.2 ± 0.1

Supplementary Table 1. Thermodynamic and kinetic parameters from analysis of C137S NMR data.

(**A**) **Determination of thermodynamic and kinetic parameters from CPMG experiments.** The asterisk (*) next to pH 6.8 for the pressure titration data indicates that this sample was prepared in 30 mM sodium phosphate buffer with 2 mM EDTA at pH 6.8. This buffer experiences a relatively large change in pH with pressure, and the the final pH is approximately 0.9 units lower at 2500 bar. The sample at pH 7 was prepared in a baroresistant buffer[20] that experiences negligible changes in pH due to increased pressure. The label "ox" refers to oxidised cHSP27. Errors are derived from fitting (k_{ex}, p_E) and propagation (k_{eg}, k_{on}, k_{off}, K_d).

(**B**) **Determination of thermodynamic and kinetic entropies and enthalpies from CPMG data.** These values were derived from fitting CPMG RD data recorded on C137S at multiple temperatures and concentrations (Methods). Errors are derived from a bootstrap analysis.

(**C**) **Determination of K_D values from HSQC spectra obtained with varying total concentration.** These values were derived from analysis of HSQC spectra recorded on C137S as a function of concentration. K_d values were calculated at each concentration where separate monomer and dimer peaks were observed, and the calculated mean and SD are indicated. The sample conditions are listed. At pH 5, no dimer peak was observed and so the K_d is only estimated.

(**D**) **Determination of volume changes with pressure and the unfolding equilibrium constant.** Samples of C137S were analysed by high-pressure NMR spectroscopy and the intensities of monomer, dimer, and unfolded monomer peaks were fit to a three-state model of unfolding. The reported errors are derived from a bootstrap analysis. Sample conditions are listed; the asterisk next to pH 6.8 refers to the same information as (A).

Residue	^{15}N $\|\Delta\omega\|$ (ppm)	^{15}N $\|\omega_{Dimer}-\omega_{RC}\|$ (ppm)
V101	2.05 ± 0.10	1.50 ± 0.68
D107	1.31 ± 0.12	9.94 ± 0.65
T110	1.24 ± 0.11	1.09 ± 1.24
D115	1.53 ± 0.07	2.88 ± 1.00
G116	0.86 ± 0.09	3.51 ± 0.50
V117	0.91 ± 0.12	0.55 ± 0.58
V118	1.65 ± 0.08	0.94 ± 0.82
T121	1.11 ± 0.08	3.77 ± 0.82
G122	3.09 ± 0.10	0.60 ± 0.84
H124	4.26 ± 0.11	2.24 ± 0.60
E125	6.19 ± 0.26	0.71 ± 1.18
E126	1.76 ± 0.09	0.10 ± 1.02
R127	4.31 ± 0.27	2.90 ± 1.21
D129	6.43 ± 0.27	6.80 ± 0.98
E130	4.31 ± 0.14	4.55 ± 0.52
G132	1.01 ± 0.41	1.64 ± 1.27
Y133	0.62 ± 0.21	1.98 ± 0.84
I134	6.26 ± 0.22	1.41 ± 0.96
S135	4.79 ± 0.23	2.11 ± 0.66
R136	8.23 ± 4.4	7.22 ± 1.71
S137	4.79 ± 0.27	3.61 ± 1.17
F138	1.39 ± 0.11	2.50 ± 0.91
T139	2.79 ± 0.18	0.82 ± 1.12
R140	3.12 ± 0.09	6.36 ± 1.03
K141	1.26 ± 0.07	1.05 ± 0.93
T143	1.01 ± 0.08	4.06 ± 1.19

Supplementary Table 2. **^{15}N $|\Delta\omega|$ values from CPMG RD analysis of C137S**. Residues in C137S that were used to fit ^{15}N CPMG RD data at 25 °C and 1 mM, their ^{15}N chemical shift changes derived from fitting the data, and predicted ^{15}N chemical shift change expected upon formation of a random coil. The ncIDP database[23] was used to determine the random coil chemical shifts. A global fit yields a reduced χ^2 value of 1.43. The fit yields the global parameters of k_{ex} and p_E 1058 ± 48 s^{-1} and of 1.70 ± 0.04 % respectively.

Residue	^{15}N $\|\Delta\omega\|$ (ppm)
D129	5.74 ± 0.53
E130	3.17 ± 0.31

Supplementary Table 3. ^{15}N $|\Delta\omega|$ values from CPMG RD analysis of $L_{5,6+7}$ in oxidised cHSP27. Residues in $L_{5,6+7}$ in oxidised cHSP27 with ^{15}N CPMG RD data and the fitted values. A global fit yields a reduced χ^2 value of 1.01. k_{ex} and p_E values of 1233 ± 291 s^{-1} and 0.82 ± 0.07 % were obtained, respectively.

Residue	^{15}N |Δω| (ppm)
R136	1.73 ± 14.9
C137	0.91 ± 7.4
F138	0.86 ± 6.6

Supplementary Table 4. ^{15}N |Δω| values from CPMG RD analysis of residues near the disulphide bond in oxidised cHSP27. Residues in oxidised cHSP27 near the intermolecular disulphide bond with ^{15}N CPMG RD data, which were fit as a group. A global fit yields a reduced χ^2 value of 1.02. The ^{15}N chemical shifts are very poorly defined with large uncertainties as the residues are approaching fast exchange and will be correlated with k_{ex} and p_E. k_{ex} and p_E values of 3210 ± 1798 s^{-1} and 0.83 ± 1.5 % were obtained respectively. Fitting all residues (Supplementary Table 3, Supplementary Table 4) in oxidised cHSP27 with one set of global parameters resulted in a poor fit.

Residue	^{15}N |Δω| (ppm)
V101	0.88 ± 0.18
D107	0.73 ± 0.17
T110	0.71 ± 0.14
D115	0.90 ± 0.29
G116	0.56 ± 0.18
V118	1.09 ± 0.11
T121	1.61 ± 0.10
G122	2.71 ± 0.17
K123	0.71 ± 0.14
H124	2.68 ± 0.16
E125	3.74 ± 0.33
E126	0.88 ± 0.12
R127	2.36 ± 0.15
D129	4.50 ± 0.41
E130	2.63 ± 0.30
Y133	0.64 ± 0.16
I134	4.07 ± 0.35
S135	2.65 ± 0.18
F138	2.98 ± 0.22
T139	2.11 ± 0.15
R140	2.34 ± 0.13
K141	1.02 ± 0.10
T143	0.71 ± 0.14

Supplementary Table 5. ^{15}N |Δω| values from CPMG RD analysis of reduced cHSP27. Residues from reduced cHSP27 that were used to fit ^{15}N CPMG RD data at 25 °C and 1 mM and their ^{15}N chemical shift changes derived from fitting the data. Note for this data, CPMG RD curves were only obtained at only one magnetic field strength. A global fit yields a reduced χ^2 value of 1.45. The fit yields the global parameters of k_{ex} and p_E 1538 ± 93 s^{-1} and of 2.33 ± 0.2 % respectively. Note that CPMG RD data were only available at one static magnetic field strength (14.1 T).

cHSP27 construct	Primer	Oligo sequence
C137S	C137S_f	CATGGCTACATCTCCCGGAGCTTCACGCGGAAATAC
C137S	C137S_r	GTATTTCCGCGTGAAGCTCCGGGAGATGTAGCCATG
H124K/C137S	H124K/C137S_f	AGATCACCGGCAAGAAAGAGGAGCGGCAGGA
H124K/C137S	H124K/C137S_r	TCCTGCCGCTCCTCTTTCTTGCCGGTGATCT

Supplementary Table 6. **Primers for site-directed mutagenesis.** DNA sequences of primers for site-directed mutagenesis of cHSP27. The construct C137S refers to residues 84-171 of HSP27 with the mutation C137S, and H124K/C137S to the same residue region with H124K and C137S mutations.

Supplementary References

1. Alderson, T. R., Benesch, J. L. P. & Baldwin, A. J. Proline isomerization in the C-terminal region of HSP27. *Cell Stress Chaperones* 1–13 (2017). doi:10.1007/s12192-017-0791-z

2. Lindner, R. a. *et al.* Mouse Hsp25, a small heat shock protein. The role of its C-terminal extension in oligomerization and chaperone action. *Eur. J. Biochem.* **267,** 1923–1932 (2000).

3. Stengel, F. *et al.* Dissecting Heterogeneous Molecular Chaperone Complexes Using a Mass Spectrum Deconvolution Approach. *Chem. Biol.* **19,** 599–607 (2012).

4. Kay, L. E., Torchia, D. A. & Bax, A. Backbone dynamics of proteins as studied by 15N inverse detected heteronuclear NMR spectroscopy: application to staphylococcal nuclease. *Biochemistry* **28,** 8972–9 (1989).

5. Farrow, N. A. *et al.* Backbone Dynamics of a Free and a Phosphopeptide-Complexed Src Homology 2 Domain Studied by 15N NMR Relaxation. *Biochemistry* **33,** 5984–6003 (1994).

6. Farrow, N. A., Zhang, O., Szabo, A., Torchia, D. A. & Kay, L. E. Spectral density function mapping using 15N relaxation data exclusively. *J. Biomol. NMR* **6,** 153–62 (1995).

7. Treweek, T. M., Rekas, A., Walker, M. J. & Carver, J. A. A quantitative NMR spectroscopic examination of the flexibility of the C-terminal extensions of the molecular chaperones, αA- and αB-crystallin. *Exp. Eye Res.* **91,** 691–699 (2010).

8. Carver, J. a, Esposito, G., Schwedersky, G. & Gaestel, M. 1H NMR spectroscopy reveals that mouse Hsp25 has a flexible C-terminal extension of 18 amino acids. *FEBS Lett.* **369,** 305–310 (1995).

9. Baldwin, A. J. *et al.* Quaternary Dynamics of αB-Crystallin as a Direct Consequence of Localised Tertiary Fluctuations in the C-Terminus. *J. Mol. Biol.* **413,** 310–320 (2011).

10. Sattler, M., Schleucher, J. & Griesinger, C. Heteronuclear multidimensional NMR experiments for the structure determination of proteins in solution employing pulsed field gradients. *Prog. Nucl. Magn. Reson. Spectrosc.* **34,** 93–158 (1999).

11. Rajagopal, P., Liu, Y., Shi, L., Clouser, A. F. & Klevit, R. E. Structure of the α-crystallin domain from the redox-sensitive chaperone, HSPB1. *J. Biomol. NMR* **63,** 223–8 (2015).

12. Hochberg, G. K. a. *et al.* The structured core domain of B-crystallin can prevent amyloid fibrillation and associated toxicity. *Proc. Natl. Acad. Sci.* **111,** E1562–E1570 (2014).

13. Cierpicki, T. & Otlewski, J. Amide proton temperature coefficients as hydrogen bond indicators in proteins. *J. Biomol. NMR* **21,** 249–61 (2001).

14. Lipari, G. & Szabo, A. Model-free approach to the interpretation of nuclear magnetic resonance relaxation in macromolecules. 1. Theory and range of validity. *J. Am. Chem. Soc.* **104,** 4546–4559 (1982).

15. Tjandra, N., Feller, S. E., Pastor, R. W. & Bax, A. Rotational diffusion anisotropy of human ubiquitin from 15N NMR relaxation. *J. Am. Chem. Soc.* **117,** 12562–12566 (1995).

16. Mandel, A. M., Akke, M. & Palmer, A. G. Backbone dynamics of Escherichia coli ribonuclease HI: correlations with structure and function in an active enzyme. *J. Mol. Biol.* **246,** 144–63 (1995).

17. Vallurupalli, P., Hansen, D. F. & Kay, L. E. Structures of invisible, excited protein states by relaxation dispersion NMR spectroscopy. *Proc. Natl. Acad. Sci.* **105,** 11766–11771 (2008).

18. Grey, M. J., Wang, C. & Palmer, A. G. Disulfide Bond Isomerization in Basic Pancreatic Trypsin Inhibitor: Multisite Chemical Exchange Quantified by CPMG Relaxation Dispersion and Chemical Shift Modeling. *J. Am. Chem. Soc.* **125,** 14324–14335 (2003).

19. Bouvignies, G. *et al.* Solution structure of a minor and transiently formed state of a T4 lysozyme mutant. *Nature* **477,** 111–114 (2011).

20. Quinlan, R. J. & Reinhart, G. D. Baroresistant buffer mixtures for biochemical analyses. *Anal. Biochem.* **341,** 69–76 (2005).

21. Alderson, T. R., Lee, J. H., Charlier, C., Ying, J. & Bax, A. Propensity for cis-Proline Formation in Unfolded Proteins. *ChemBioChem* **19,** 37–42 (2018).

22. Han, B., Liu, Y., Ginzinger, S. W. & Wishart, D. S. SHIFTX2: significantly improved protein chemical shift prediction. *J. Biomol. NMR* **50,** 43–57 (2011).

23. Tamiola, K., Acar, B. & Mulder, F. A. A. Sequence-specific random coil chemical shifts of intrinsically disordered proteins. *J. Am. Chem. Soc.* **132,** 18000–3 (2010).

24. Rajagopal, P. *et al.* A conserved histidine modulates HSPB5 structure to trigger chaperone activity in response to stress-related acidosis. *Elife* **4,** 1–21 (2015).
